# Supplementary material for: Genomic Correlates of DNA Damage in Breast Cancer Subtypes
Source: Cancers (Basel). 2021 Apr 27;13(9):2117. doi: 10.3390/cancers13092117 (PMC8123819; doi:10.3390/cancers13092117)
Supplement: Supplementary file 1 [file cancers-13-02117-s001.zip › cancers-1176188-supplementary.pdf]

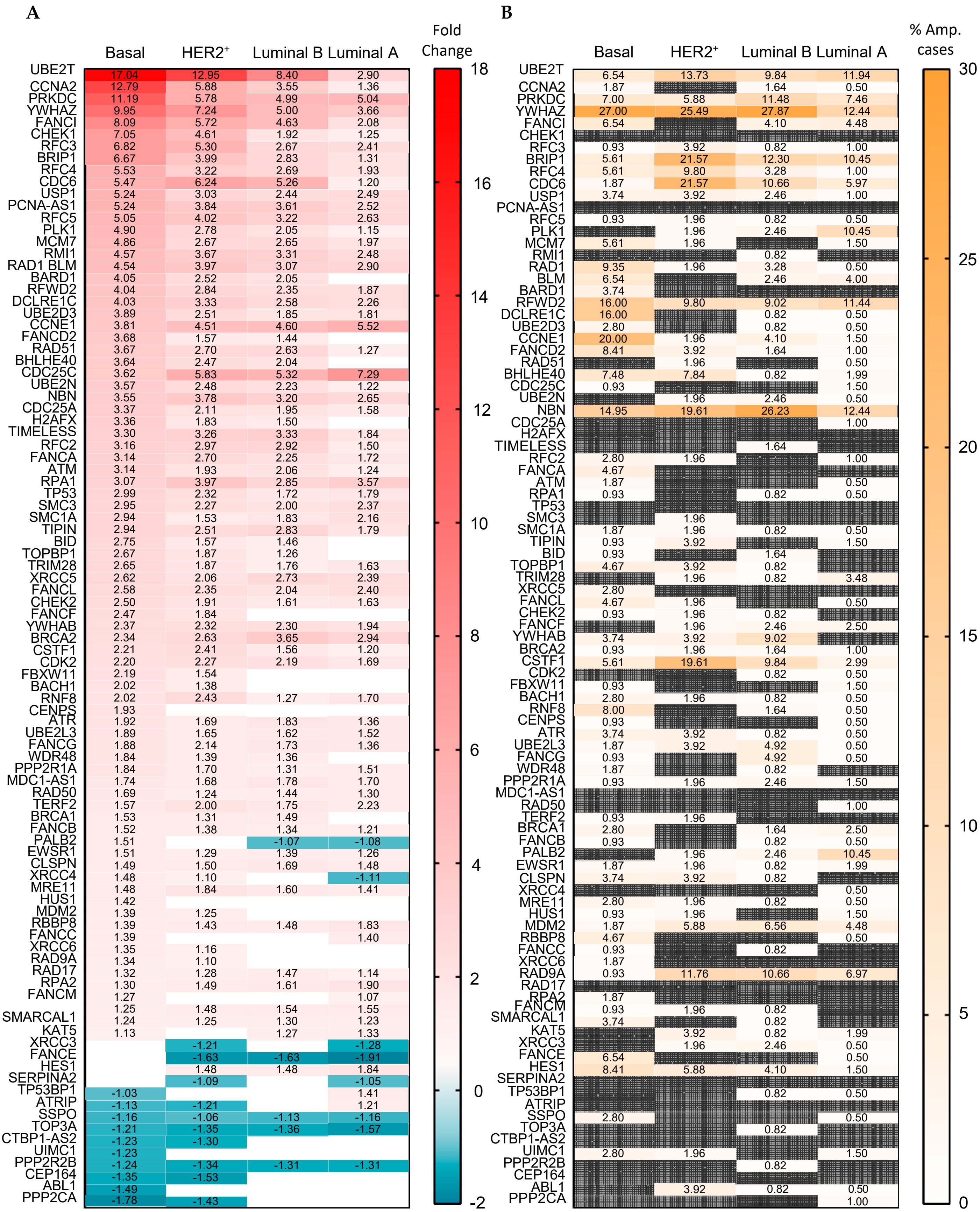

Figure S1: Upregulation of most of the genes involved in the DNA damage gene sets in BC

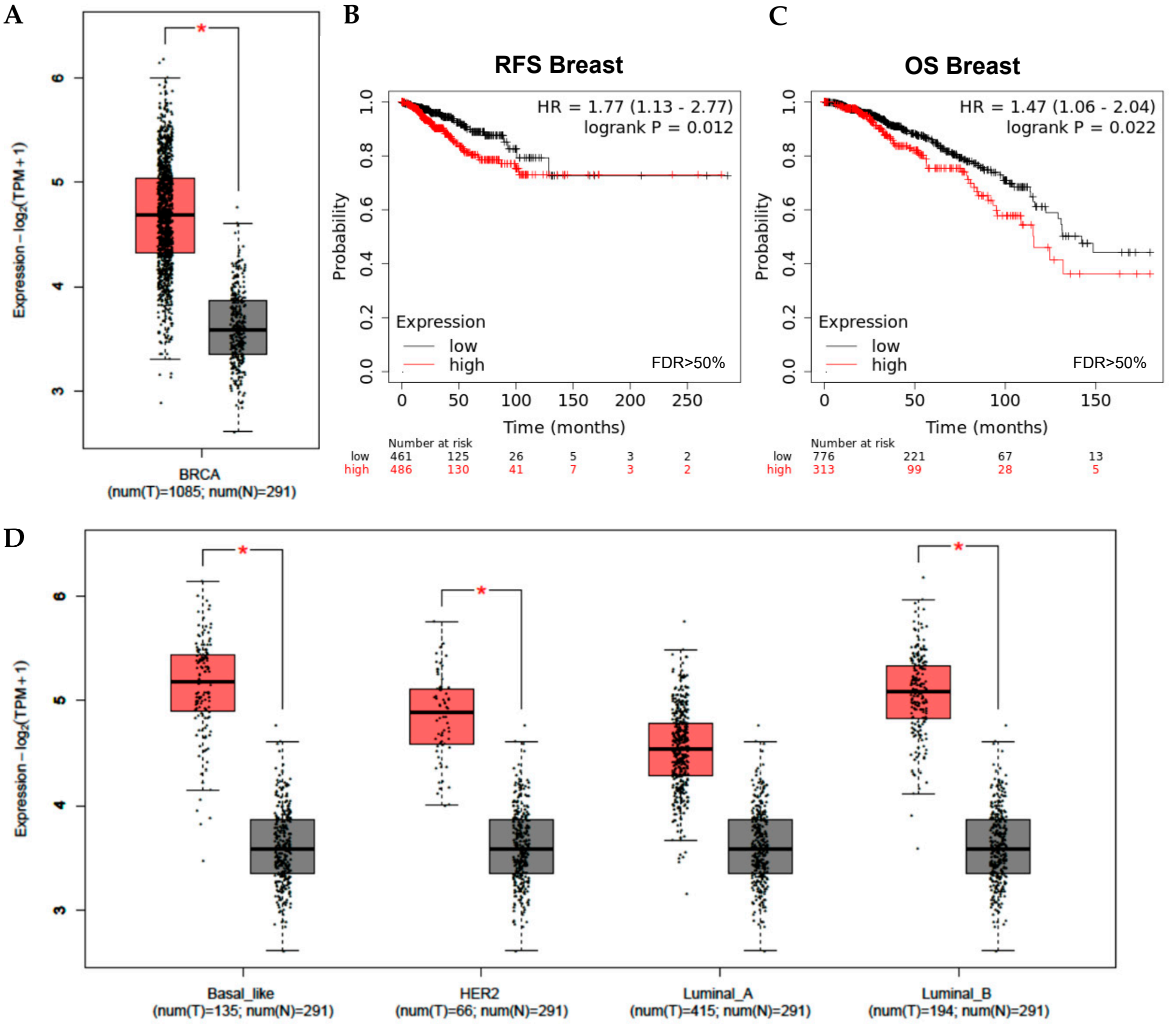

Figure S2: Upregulated genes in all breast cancer subtypes correlates with poor breast cancer survival.

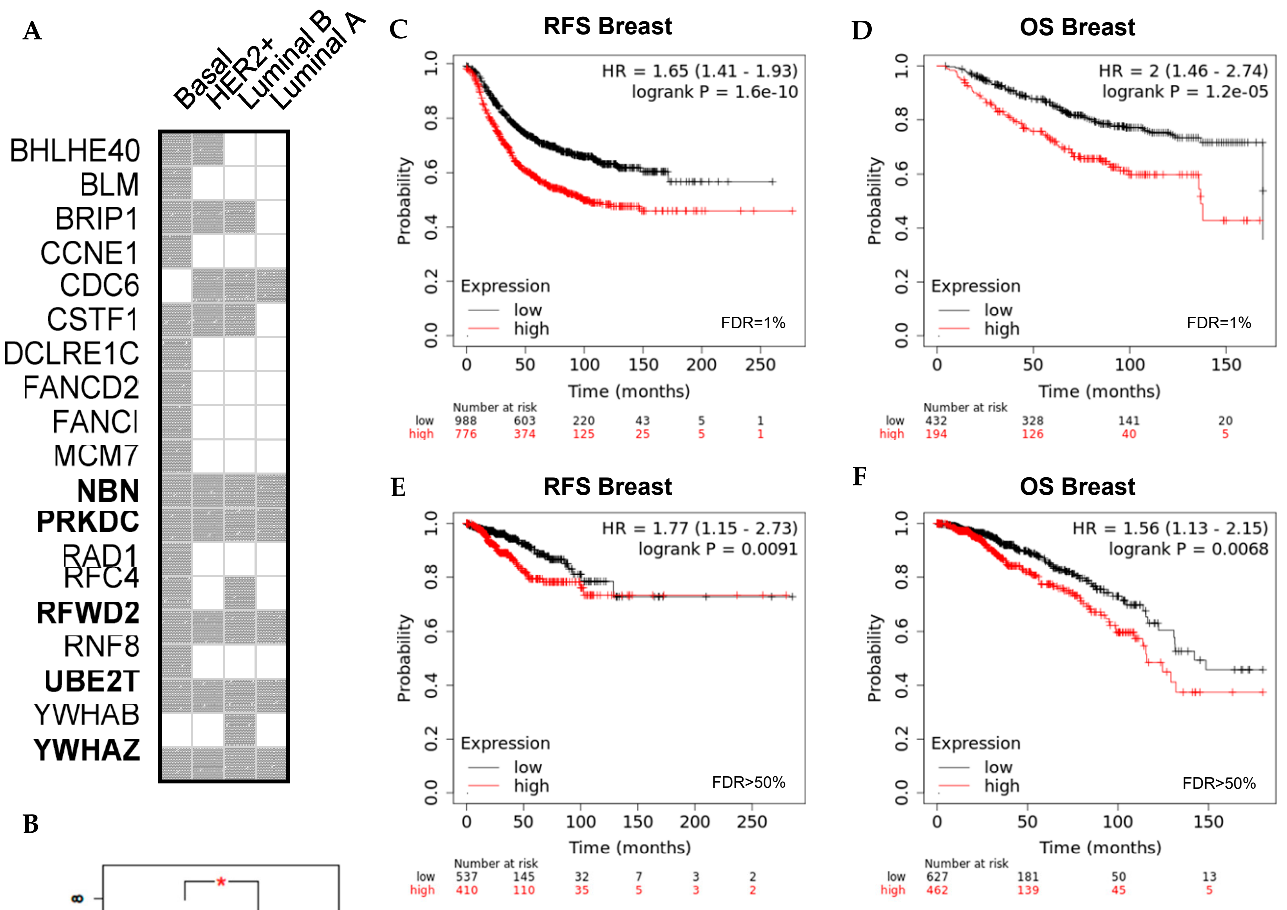

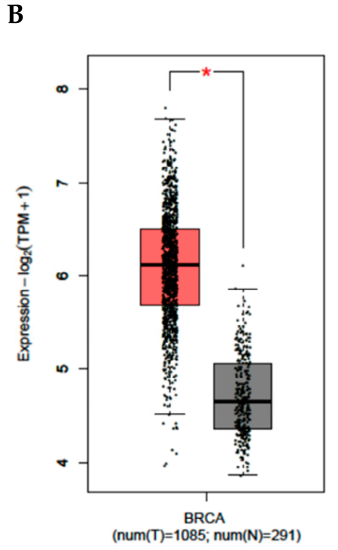

G

Gene	Function
NBN	Component of the MRN involved in DNA damage response chromosome integrity maintenance
PRKDC	Serine/threonine-kinase involved in NHEJ required for DSB re and V(D)J recombination
RFWD2	E3 ubiquitin-protein ligase that leads to leading to AKT activa and promotion of cell survival.
UBE2T	Acts as a specific E2 ubiquitin-conjugating enzyme for the Fan anemia complex, a key step in the DNA damage pathway
YWHAZ	Adapter protein implicated in the regulation of a large spectrur both general and specialized signaling pathways.

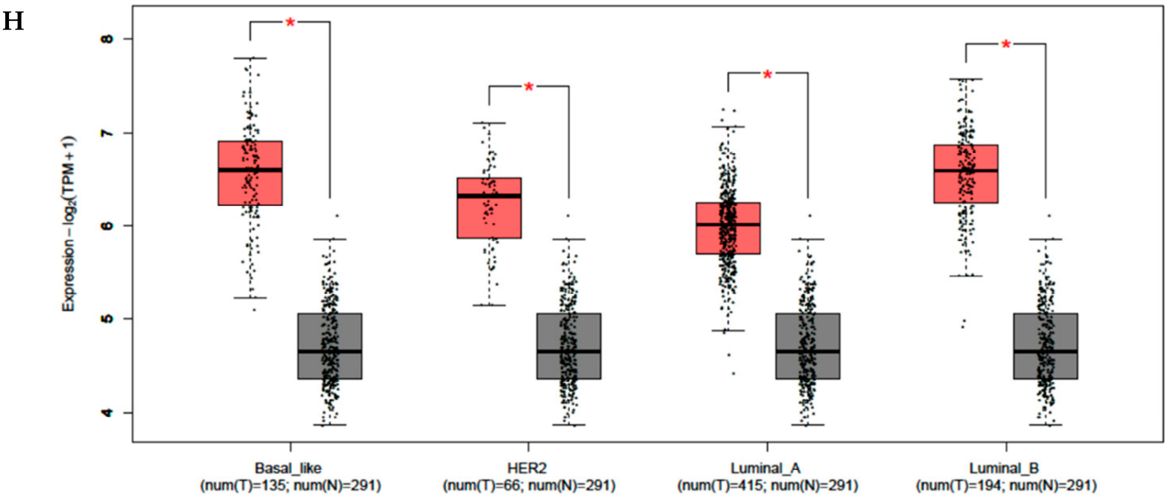

Figure S3: Upregulated 5-genes signature in all breast cancer subtypes correlates with poor breast cancer survival
